# Further Spread of a *bla*_KPC_-Harboring Untypeable Plasmid in *Enterobacteriaceae* in China

**DOI:** 10.3389/fmicb.2018.01938

**Published:** 2018-08-21

**Authors:** Jiansheng Huang, Hui Ding, Yang Shi, Yunan Zhao, Xiaolei Hu, Jianmin Ren, Guiying Huang, Rongzhen Wu, Zhigang Zhao

**Affiliations:** ^1^Lishui Hospital, Zhejiang University School of Medicine, Lishui, China; ^2^The Fifth Affiliated Hospital of Wenzhou Medical University, Lishui, China; ^3^Lishui Municipal Central Hospital, Lishui, China

**Keywords:** *Enterobacteriaceae*, carbapenemase, *bla*_KPC_, untypeable plasmid, dissemination

## Abstract

The wide spread of *Klebsiella pneumoniae* carbapenemase (KPC)-producing *Enterobacteriaceae* is great threat to public health in China. Plasmids are among the major factors mediating *bla*_KPC_ gene dissemination. A total of 156 carbapenem-resistant *Enterobacteriaceae* (CRE) isolates were identified in a tertiary hospital in China. Six KPC-producing isolates, namely, *E. coli* (*n* = 2), *E. asburiae* (*n* = 1), *C. freundii* (*n* = 1), *C. portucalensis* (*n* = 1), and *C. koseri* (*n* = 1), tested positive for the pCKPC18-1-like untypeable plasmid, which was described recently in *C. freundii*. All 6 plasmids could be easily transferred into *E. coli* by chemical transformation or conjugation and were confirmed by sequencing to harbor *bla*_KPC−2_. Multilocus PCRs and EcoRI-RFLP revealed that the 6 untypeable plasmids belonged to 2 isoforms. High-throughput sequencing of representative plasmids (pCP40 and pEC86) led to the identification of 2 plasmids that shared the common backbone genes *repA, DnaJ, StpA*, and *yafB*, which were characteristic of the untypeable plasmid, and had similar *bla*_KPC−2_ genetic contexts of the Tn*3*-Tn*4401* chimera. Nucleotide comparison revealed high sequence identity of the 2 plasmids with previously reported *bla*_KPC−2_-carrying untypeable plasmids. In particular, the pCP40 plasmid from *C. portucalensis* and the pHS062105-3 plasmid from *K. pneumoniae* differed by only 20 single-nucleotide polymorphisms (SNPs). To the best of our knowledge, this is the first report of a *bla*_KPC_-harboring untypeable plasmid spread into *E. coli, E. asburiae*, and *C. koseri* strains in China.

## Introduction

Carbapenems are often used as the last effective agents in the treatment of severe infections caused by multidrug-resistant gram-negative bacteria, especially strains expressing high-level AmpC cephalosporinase or extended spectrum β-lactamases (ESBLs). However, the emergence of carbapenem-hydrolyzing enzymes has greatly limited the effectiveness of these agents (Nordmann et al., 2009; Zhang et al., 2017).

*Klebsiella pneumoniae* carbapenemase (KPC), an Ambler class A enzyme, is a powerful carbapenem-hydrolyzing enzyme and can hydrolyze most of the beta-lactams (Nordmann et al., 2009). Since the first report of the *bla*_KPC_ gene from *Klebsiella pneumoniae* isolated in North Carolina (USA) in 2001 (Yigit et al., 2001), this gene has been identified in multiple genera and species of *Enterobacteriaceae*, including *K. pneumoniae* (Naas et al., 2008; Gootz et al., 2009; Monteiro et al., 2009; Shen et al., 2009), *Escherichia coli* (Cai et al., 2008; Chen L. et al., 2014), *Salmonella* spp. (Cai et al., 2008), and *Serratia marcescens* (Zhang et al., 2007; Cai et al., 2008), and has even been identified in nonfermenters such as *Acinetobacter* (Robledo et al., 2010) and *Pseudomonas* spp. (Poirel et al., 2010). Rapid spread of the *bla*_KPC_ gene is of great concern (Nordmann et al., 2009; Zhang et al., 2017).

Plasmids play an important role in the dissemination of the *bla*_KPC−2_ gene. Only one identified *bla*_KPC−18_ gene has been determined to be located on the chromosome (Thomson et al., 2016), while nearly all of the other identified *bla*_KPC_ genes were harbored on plasmids. Plasmids of the incompatibility groups F (IncF), N (IncN), L/M (IncL/M), and X (IncX) have been reported to mediate *bla*_KPC−2_ gene transfer in *Enterobacteriaceae* (Cuzon et al., 2010; Jiang et al., 2010; Chen et al., 2013; Chen L. et al., 2014; Chen Y. T. et al., 2014).

Recently, a novel *bla*_KPC_-harboring untypeable plasmid (pCKPC18-1) encoding a replication protein that could not be assigned to any known incompatibility group was described in China (Zheng et al., 2018). To date, the likely plasmid has only been detected in *Citrobacter freundii, Klebsiella pneumonia*, and *Enterobacter cloacae* strains (Jiang et al., 2015; Shen et al., 2016; Zheng et al., 2018). In this study, we provided evidence for the further spread of the *bla*_KPC_-carrying untypeable plasmid in *Enterobacteriaceae* in the southwestern Zhejiang province of China.

## Materials and methods

### Bacterial strains, detection of carbapenem resistance genes, and plasmid incompatibility typing

From 2011 to 2016, a total of 156 non-repetitive carbapenem-resistant *Enterobacteriaceae* (CRE) strains were isolated by the VITEK2 Compact system (bioMérieux VITEK, USA) in Lishui Hospital of Zhejiang University, a tertiary hospital in southwestern Zhejiang province of China. Species were further identified using an automated mass spectrometry microbial identification system (MALDI-TOF, Bruker, USA) and re-confirmed by 16S rRNA, *RecN*, and *Hsp60* sequencing (Hoffmann and Roggenkamp, 2003; Ribeiro et al., 2015). Common carbapenemase-encoding genes, including *bla*_KPC_, *bla*_NDM_, *bla*_*VIM*_*, bla*_*IMP*_*, bla*_GES_, *bla*_SME_, *bla*_IMI_, *bla*_SIM_, *bla*_GIM_, *bla*_SPM_, *bla*_OXA−23_*, bla*_OXA−24_, and *bla*_OXA−58_, from all 156 isolates were amplified, and amplicons were sequenced (Pfeifer et al., 2010; Huang et al., 2012; Zhang et al., 2017, 2018). Plasmids of the untypeable incompatibility group, represented by pCKPC18-1 (CP022276), were screened by specific primers (UTF-TGCATCGATACGTTCCTGCA and UTR-ACTCGCTAGCATGGAACATC) targeting the replication initiator (*repA*). The untypeable *repA*-positive isolates were selected for further studies.

### Antimicrobial susceptibility testing

Antimicrobial susceptibilities were first determined by the disc diffusion (Kirby-Bauer, K-B) method. The minimum inhibitory concentrations (MICs) of imipenem, ertapenem, ceftazidime, ceftriaxone, cefepime, ampicillin, aztreonam, ciprofloxacin, levofloxacin, trimethoprim/sulfamethoxazole, tobramycin, gentamicin, and amikacin were detected by the VITEK2 Compact system with AST-GN13 cards or *E*-test (bioMérieux, France) according to the manufacturer's instructions. The results were interpreted according to the guidelines of the Clinical and Laboratory Standards Institute (CLSI) (Wayne, 2017). *E. coli* ATCC 25922 was used for quality control.

### Determination of genetic relatedness

Multilocus sequence typing was performed on the *E. coli* (http://mlst.warwick.ac.uk/mlst/dbs/Ecoli) and *C. freundii* (https://pubmlst.org/cfreundii/) isolates according to the online databases. Pulsed-field gel electrophoresis (PFGE) was performed to further evaluate the relatedness of the *E. coli* strains via XbaI digestion (Huang et al., 2016).

### Multilocus PCR and RFLP analysis of plasmids

Transformation and conjugation experiments were performed to acquire purified single plasmids as described previously (Jiang et al., 2010, 2015; Shen et al., 2016; Zheng et al., 2018). The *bla*_KPC−2_-carrying plasmids were extracted with a Plasmid Miniprep Kit (Transgen Biotech, China) from the transconjugants. Multilocus PCR was performed to evaluate the relationships of the 6 untypeable plasmids discovered in this study. In addition to the *repA* and *bla*_KPC_ genes, primers (Table 1) were designed to target the backbone genes *taxA, virB5, virB11*, and *repB* and the mobile elements Tn*1721*-*TnpA* and Tn*1721*-*TnpR*. Amplicons were analyzed by electrophoresis and sequencing. Meanwhile, plasmids were digested with EcoRI and subjected to restriction fragment length polymorphism (RFLP) by electrophoresis on 1% agarose (Sangon, China) gels in 1 × TAE buffer as reported before (Ho et al., 2012).

**Table 1:** Multilocus-PCR primers used in this study.

**Target**	**Primer**	**Sequence**	**Size (bp)**	**References**
*repA*	UTF	TGCATCGATACGTTCCTGCA	841	pCKPC18-1 and pFOS18
	UTR	ACTCGCTAGCATGGAACATC		
*bla*_KPC_	KPCF	GCTACACCTAGCTCCACCTTC	989	Shen et al., 2009
	KPCR	ACAGTGGTTGGTAATCCATGC		
*taxA*	CH1F	GCGTTGAATCCACGTATTGG	752	pCKPC18-1
	CH1R	TATCATGCCCGTATACTCGC		
*virB5*	CH2F	GCACCTTGTGGTGAAGAACC	779	pCKPC18-1
	CH2R	TGTACGGCATTAGCGGCATC		
*virB11*	CH3F	CTACGTTCGTCAATTCACTG	772	pCKPC18-1
	CH3R	AGGTGTAGATCACCAACGCG		
Tn*1721*-*TnpA*	CH4F	CAGGTAGTCGTCGAAGTCGC	815	pCKPC18-1
	CH4R	CCAACTCTCGGCACATGCTG		
Tn*1721*-*TnpR*	CH5F	TCTGTACCAAGCGACGCAGG	762	pCKPC18-1
	CH5R	CGGCCTCATGGTACATCTGG		
*TnpA*/*repB*	CH4F	CAGGTAGTCGTCGAAGTCGC	954 or 2558	pCKPC18-1 and pFOS18
	CH5R	CGGCCTCATGGTACATCTGG		

### Plasmid sequencing and annotation

Representative plasmids were fragmented by the whole-genome shotgun (WGS) approach and libraries were constructed. Genomic DNA were completely sequenced by next-generation sequencing (NGS) on an Illumina MiSeq platform with 2 × 400 bp paired-end reads. Adapters were removed using AdapterRemoval (ver. 2.1.7), and the high quality reads were screened through SOAPec (v2.0) with a Kmer frequency of 17. Sequences were then assembled with the A5-miseq (v20160825) and SPAdes (v3.9.0) programs. Protein-coding genes were predicted using GeneMarkS (v4.28) software, and coding sequence (CDS) annotations were performed using the BLASTP program with the NR database, followed by manual inspection.

## Results

### Bacterial strains and antimicrobial susceptibility testing

Among all the 156 strains, 6 *bla*_KPC−2_ producers [2 *E. coli* strains (EC84 and EC86), 1 *E. asburiae* strain (EAK7), 1 *C. freundii* strain (CF111), 1 *C. portucalensis* strain (CP40), and 1 *C. koseri* strain (CK61)] isolated from different patients were positive for the untypeable plasmid (Supplementary S1). EAK7, which was isolated from hematology wards in December 2010, was the first untypeable plasmid carrier identified in our hospital. Then it was CP40 in January 2012, CK61 in September 2012, EC84 in May 2013, EC86 in June 2013 and CF111 in October 2014. However, except for the 2 *E. coli* strains isolated from the ICU, all the strains were isolated from different wards. No apparent contact among the patients was validated. None of the other CRE isolates were positive for the untypeable plasmid. All 6 isolates were resistant to carbapenems, third-generation cephalosporins and aztreonam, but were susceptible to colistin (Table 2). Notably, *C. portucalensis* CP40 was resistant to sulfonamides and aminoglycosides but was susceptible to quinolones; however, *C. freundii* CF111 was exhibited the opposite resistance and susceptibility. Both *E. coli* EC84 and EC86 were resistant to levofloxacin, ciprofloxacin, trimethoprim/sulfamethoxazole, gentamicin and tobramycin and were susceptible to amikacin. Nevertheless, all the transconjugants showed similar susceptibility profiles that were resistant to the β-lactams but susceptible to quinolones, aminoglycosides and sulfonamides.

**Table 2:** Antimicrobial susceptibilities of the six enterobacteriaceae and their transconjugants.

**Isolates**	**Species**	**IPM**		**ETP**		**FEP**		**CAZ**		**CRO**		**AMP**		**ATM**		LEV*		CIP*		**SXT**		**Gm**		**TM**		**AMK**		**PB**
		**MIC**	**KB**	**MIC**	**KB**	**MIC**	**KB**	**MIC**	**KB**	**MIC**	**KB**	**MIC**	**KB**	**MIC**	**KB**	**MIC**	**KB**	**MIC**	**KB**	**MIC**	**KB**	**MIC**	**KB**	**MIC**	**KB**	**MIC**	**KB**	**KB**
EAK7	*E. asburiae*	≥16	R	≥8	R	≥64	R	8	R	≥64	R	≥32	R	≥64	R	1	S	0.5	S	≤ 20	S	≥16	R	≥16	R	≥64	R	S
CP40	*C. portucalensis*	≥16	R	≥8	R	32	R	≥64	R	≥64	R	≥32	R	≥64	R	8	R	4	R	≤ 20	S	≤ 1	S	≤ 1	S	≤ 2	S	S
CK61	*C. koseri*	2	I	8	R	16	R	16	R	32	R	≥32	R	≥64	R	0.064	S	0.016	S	≤ 20	S	8	R	≤ 1	S	≤ 2	S	S
EC84	*E. coli*	8	R	≥8	R	≥64	R	≥64	R	≥64	R	≥32	R	≥64	R	≥32	R	≥32	R	≥320	R	≥16	R	8	I	≤ 2	S	S
EC86	*E. coli*	8	R	≥8	R	≥64	R	≥64	R	≥64	R	≥32	R	≥64	R	≥32	R	≥32	R	≥320	R	≥16	R	≥16	R	4	S	S
CF111	*C. freundii*	≥16	R	≥8	R	≥64	R	16	R	≥64	R	≥32	R	≥64	R	2	S	1	I	≥320	R	≥16	R	≥16	R	≥64	R	S
Tc-K7	*E. coli* J53	≥16	R	≥8	R	≥64	R	≥64	R	≥64	R	≥32	R	≥64	R	0.064	S	0.016	S	≤ 20	S	4	S	≤ 1	S	4	S	S
Tc-40	*E. coli* DH5α	≥16	R	≥8	R	≥64	R	≥64	R	≥64	R	≥32	R	≥64	R	0.008	S	0.008	S	≤ 20	S	2	S	2	S	4	S	S
Tc-61	*E. coli* J53	≥16	R	≥8	R	≥64	R	≥64	R	≥64	R	≥32	R	≥64	R	0.064	S	0.016	S	≤ 20	S	4	S	2	S	4	S	S
Tc-84	*E. coli* DH5α	≥16	R	≥8	R	≥64	R	≥64	R	≥64	R	≥32	R	≥64	R	0.016	S	0.008	S	≤ 20	S	2	S	2	S	≤ 2	S	S
Tc-86	*E. coli* DH5α	≥16	R	≥8	R	≥64	R	≥64	R	≥64	R	≥32	R	≥64	R	0.016	S	0.008	S	≤ 20	S	2	S	2	S	≤ 2	S	S
Tc-111	*E. coli* DH5α	≥16	R	≥8	R	≥64	R	≥64	R	≥64	R	≥32	R	≥64	R	0.008	S	0.008	S	≤ 20	S	4	S	≤ 1	S	4	S	S
J53	*E. coli* J53	≤ 1	S	≤ 0.5	S	≤ 1	S	≤ 1	S	≤ 1	S	4	S	≤ 1	S	0.064	S	0.008	S	≤ 20	S	≤ 1	S	≤ 1	S	≤ 2	S	S
Trans1-T1	*E. coli* DH5α	≤ 1	S	≤ 0.5	S	≤ 1	S	≤ 1	S	≤ 1	S	4	S	≤ 1	S	0.008	S	0.016	S	≤ 20	S	≤ 1	S	≤ 1	S	≤ 2	S	S
25922	*E. coli*	≤ 1	S	≤ 0.5	S	≤ 1	S	≤ 1	S	≤ 1	S	2	S	≤ 1	S	0.008	S	0.008	S	≤ 20	S	≤ 1	S	≤ 1	S	≤ 2	S	S

Susceptibilities were determined by KB method and MICs (μg/mL) were obtained by VITEK2 compact system with AST-GN13 cards, the results were interpreted according to CLSI guidelines. Tc, transconjugants. J53, azide-resistant E. coli J53Az^R^ strain; Trans1-T1, E. coli competent cells; 25922, E. coli ATCC25922 strain. IMP, imipenem; ETP, ertapenem; FEP, cefepime; CAZ, ceftazidime; CRO, ceftriaxone; AMP, ampicillin, ATM, aztreonam; LEV, levofloxacin; SXT, trimethoprim/sulfamethoxazole; CIP, ciprofloxacin; Gm, gentamicin; TM, tobramycin; AMK, amikacin. S, susceptible; I, intermediate; R, resistant. *Tested by E-test technique (AB bioMerieux, Sweden).

### Analysis of the genetic relatedness

According to the MLST results, both *E. coli* EC84 and EC86 belonged to ST648 and showed identical PFGE patterns (Supplementary S2), suggesting the clonal relatedness of the 2 strains. The *C. freundii* CF111 belonged to sequence type ST116.

### Multilocus PCR and RFLP analysis of plasmids

**Figure 1:**
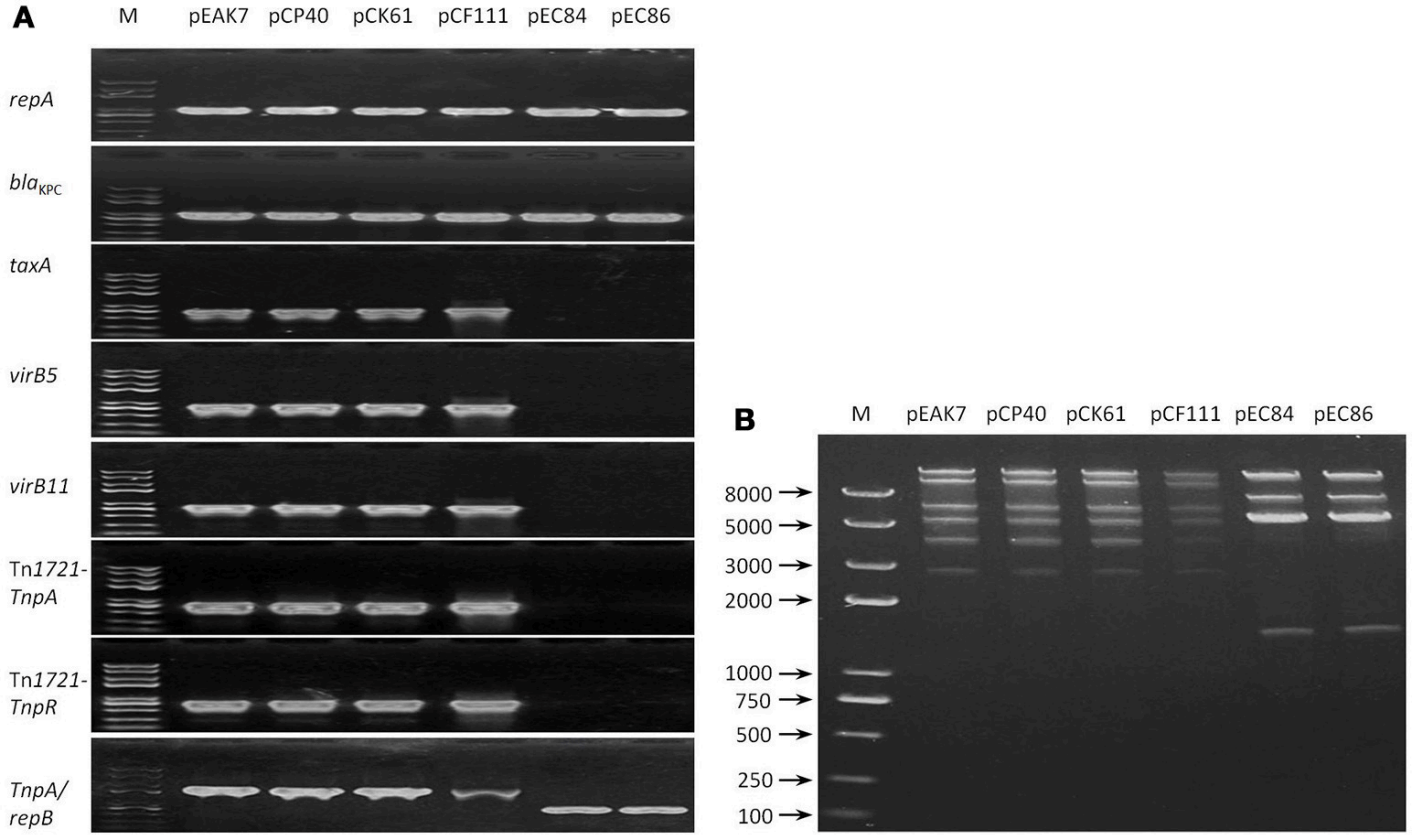
Relatedness of the 6 untypeable plasmids. **(A)** Mutilocus-PCR results. Primers targeting the genes *repA, bla*_KPC_, *TaxA, virB5, virB11*, Tn*1721*-*TnpA*, Tn*1721*-*TnpR*, and *repB* were designed. Two profiles were obtained after evaluation of the amplicons by electrophoresis. The plasmids pEAK7, pCP40, pCK61, and pCF111 exhibited one pattern, while pEC84 and pEC86 exhibited a different pattern. **(B)** RFLP results. Fragments were separated by electrophoresis on a 1% agarose gel in 1 × TAE buffer. Two different patterns, one for the plasmids pEAK7, pCP40, pCK61, and pCF111 and another for pEC84 and pEC86, were obtained. EA, *E. asburiae*; CP, *C. portucalensis*; CF, *C. freundii*; CK, *C. koseri*; EC, *E. coli*; M, DNA marker.

Single plasmids were successfully obtained via transformation (pCP40, pCF111, pEC84, and pEC86) and conjugation (pEAK7 and pCK61). All the plasmids tested positive for *repA* and *bla*_KPC_. Upon amplification of the *TaxA, virB5, virB11*, Tn*1721*-*TnpA*, and Tn*1721*-*TnpR* sequences, positive results, which exhibited 100% sequence identity with pCKPC18-1, were obtained with pEAK7, pCP40, pCK61, and pCF111; however, none of these genes were detected in pEC84 and pEC86 (Figure 1A). Interestingly, fragments of different lengths were obtained from all the plasmids with the forward and reverse primers CH4F and CH5R, targeting Tn*1721*-*TnpA* and *repB*, respectively. The fragment lengths were approximately 2,500 bp from pEAK7, pCP40, pCK61 and pCF111 and 1,000 bp from pEC84 and pEC86. Sequencing analysis revealed that the large and small fragments shared 100% identity with pCKPC18-1 (CP022276) and pKPC-ECN49 (KP726894), respectively. Consistent with the multilocus PCR results, 2 different patterns were observed in the RFLP analysis (Figure 1B). The plasmids pEAK7, pCP40, pCK61, and pCF111 yielded identical fragments, which differed from the fragments from pEC84 and pEC86.

### Characteristics of the *bla*_KPC-2_-carrying untypeable plasmids

**Figure 2:**
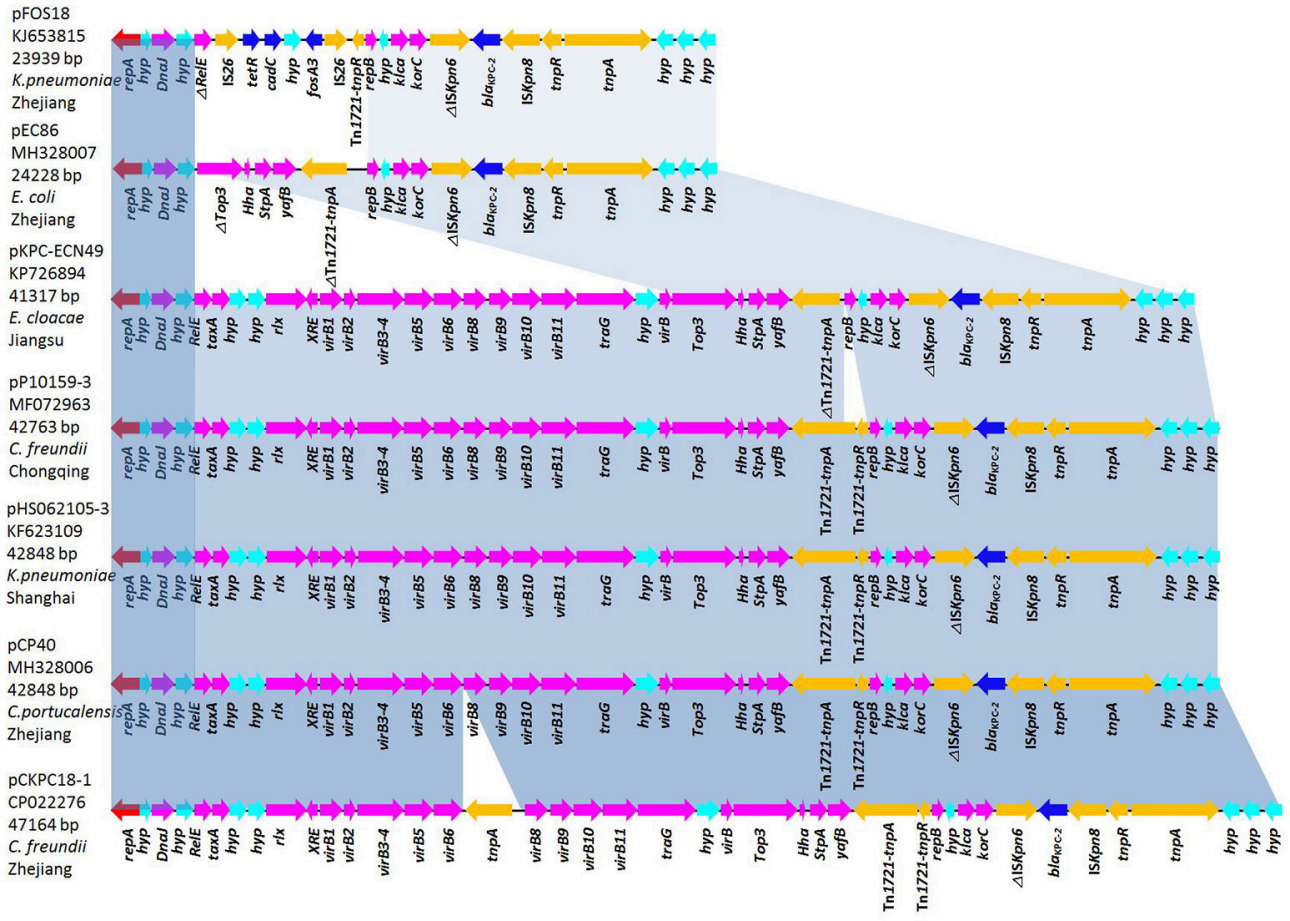
Comparative analysis of the *bla*_KPC_-harboring untypeable plasmids pFOS18 (KJ653815), pEC86 (MH328007), pKPC-ECN49 (KP726894), pHS062105-3 (KF623109), pP10159-3 (MF072963), pCP40 (MH328006), and pCKPC18-1 (CP022276). Light-blue shading denotes shared regions of homology in each adjacent plasmid. Notably, the type IV secretory pathway (VirB1-10) present in pKPC-ECN49, pHS062105-3, pP10159-3, pCP40, and pCKPC18-1 is absent in pFOS18 and pEC86. Open reading frames (ORFs) are depicted by arrows and are colored based on predicted gene function. Resistance genes are indicated with deep-blue arrows.

The plasmids pCP40 and pEC86, isolated from *C. portucalensis* and *E. coli* strains respectively, were chosen as representative plasmids for complete genome sequencing. The plasmid pCP40 is a 42,848-bp closed circular DNA with an average G+C content of 50.1%. Annotation of the final sequence of pCP40 revealed 50 open reading frames (ORFs), 32 of which encoded homologous proteins with known functions. In the backbone structure, there were genes encoding an untypeable replication protein, the molecular chaperone DnaJ, the type IV secretory pathway (VirB1-10), the DNA-binding protein StpA, the antirestriction protein Klca, and the transcriptional repressor protein KorC. In the variable region, the *bla*_KPC−2_ gene was embedded in the Tn*3*-Tn*4401* chimera with the gene order Tn*3*-*TnpA*, Tn*3*-*TnpR*, IS*Kpn8, bla*_KPC−2_, IS*Kpn6*-*like*, Tn*1721*-*TnpR*, and Tn*1721*-*TnpA* (Figure 2).

The plasmid pEC86 was 24,228-bp in length, possessed an average G+C content of 50.9% and had 37 predicted ORFs. This plasmid contained backbone genes encoding the replicons, DnaJ, StpA, Klca, and KorC, though the type IV secretory system (VirB1-10) was absent. Similar to pCP40, *bla*_KPC−2_ was the only resistance gene present in pEC86 and was integrated in the same genetic context but without Tn*1721*-*TnpR* and with a partial Tn*1721*-*TnpA* (Figure 2).

The sequences of 5 *bla*_KPC_-harboring plasmids with identical *repA* genes were downloaded from the NCBI database. pCP40 shared 99% sequence similarity with the untypeable plasmids pHS062105-3 (KF623109, 42,848-bp), pP10159-3 (MF072963, 42,763-bp), pCKPC18-1 (CP022276, 47,164-bp), and pKPC-ECN49 (KP726894, 41,317-bp). In particular, with the exception of 20 single-nucleotide polymorphisms (SNPs), pCP40 was identical to the plasmid pHS062105-3 from a *K. pneumoniae* isolate from Shanghai. The overall structure of pEC86 was most similar to that of pFOS18 (KJ653815, 23,939-bp) from *K. pneumoniae* and pKPC-ECN49 from *E. cloacae* (Jiang et al., 2015). However, the *fosA3* gene embedded the IS*26* composite transposon in pFOS18 and the type IV secretory pathway in pKPC-ECN49 was not observed in pEC86.

### Nucleotide accession numbers

The complete sequences of pCP40 and pEC86 have been deposited in GenBank under the accession numbers MH328006 and MH328007, respectively.

## Discussion

Plasmids are one of the major causes responsible for the rapid dissemination of the *bla*_KPC_ gene (Cuzon et al., 2010; Jiang et al., 2010; Chen et al., 2013; Chen L. et al., 2014; Chen Y. T. et al., 2014). The novel plasmid pFOS18, characterized by backbone genes encoding a replication protein (RepA) that could not be assigned to any known incompatibility group, the molecular chaperone DnaJ, the DNA-binding protein StpA, the antirestriction protein Klca, and the transcriptional repressor protein KorC, was first described in 2015 from a fosfomycin-resistant KPC-producing *K. pneumoniae* strain in Zhejiang province (Jiang et al., 2015). Recently, plasmids containing identical *repA* genes were detected in *Citrobacter freundii* (pCKPC18-1) and *Enterobacter cloacae* (pKPC-ECN49) stains in the Zhejiang and Jiangsu provinces, respectively (Zheng et al., 2018).

In this study, we identified the untypeable plasmids in 6 *Enterobacteriaceae*. Besides *C. freundii* strain, the untypeable plasmid was detected in 2 *E. coli*, 1 *E. asburiae*, 1 *C. portucalensis* and 1 *C. koseri* strains. Although *E. asburiae* was the first strain carrying the untypeable plasmid isolated in our hospital, clonal spread of this strain was not detected. The 2 *E. coli* strains exhibited identical ST types, PFGE profiles and susceptibility patterns, suggesting clonal spread of the *E. coli* strains. However, due to limited information, it is not clear whether the CF111 (ST116) is related to the *C. freundii* strains carrying pCKPC18-1 in Hangzhou or pP10159-3 in Chongqing (Zheng et al., 2018).

Consistent with the previous studies (Jiang et al., 2015; Shen et al., 2016; Zheng et al., 2018), 2 different subtypes of the untypeable plasmids were characterized in the 6 strains according to multilocus-PCR and RFLP analysis. The plasmids pEAK7, pCP40, pCK61, and pCF111 yielded identical multilocus-PCR results matching pCKPC18-1, while the other 2 (pEC84 and pEC86) were more related to pFOS18. Complete sequencing of the 2 representative plasmids, pCP40 and pEC86, showed that they were 42,848 and 24,228-bp in size, respectively. Although they differed in size, identical replication initiators and similar backbone genes and *bla*_KPC−2_ genetic contexts were observed in the 2 plasmids. Comparative analysis revealed that pCP40 shared high query coverage and identity with untypeable plasmids isolated from *K. pneumoniae, C. freundii* and *E. cloacae* in different areas of China. In particular, pCP40 present in the *C. portucalensis* isolate and pHS062105-3 from a *K. pneumoniae* strain differed by only 20 SNPs. Taken together, the high sequence similarity among pCP40, pKPC-ECN49, pHS062105-3, pP10159-3, and pCKPC18-1 suggested that these plasmids evolved from a common plasmid and spread independently (Zheng et al., 2018).

A previous study showed that the *K. pneumoniae* strains could transfer their *bla*_KPC−2_-carrying untypeable plasmids to the azide-resistant *E. coli* strain J53, implying the possibility of horizontal transfer of the untypeable plasmid (Shen et al., 2016). Moreover, the transfer regions of these untypeable plasmids also share high identity with the transferable IncN plasmid p1 (CP006657) (Zheng et al., 2018). In our study, 2 strains (EAK7 and CK61) successfully transferred untypeable plasmids to the recipient, thereby reconfirming the horizontal transfer capacity. Take account of the high similarity of pEAK7, pCP40, pCK61, and pCF111, the untypeable plasmid may transfer among *Enterobacteriaceae*, mediating *bla*_KPC_ dissemination. However, after 5 attempts, the *E. coli* strains (EC84 and EC86) failed in conjugation experiments. Compared with pCP40, complete sequencing revealed the main difference of lacking the type IV secretory system in pEC86. The type IV secretory system has been reported to deliver DNA and protein substrates from donor to target bacterial cells by conjugation (Christie, 2004), implying that it is the factor responsible for the horizontal transferability of the untypeable plasmid.

In summary, this study characterized the epidemiological contexts of 6 CRE isolates carrying the untypeable plasmids in our hospital and identified two plasmids, namely, pCP40 and pEC86. To the best of our knowledge, this is the first description of a *bla*_KPC_-harboring untypeable plasmid in *E. coli, E. asburiae*, and *C. koseri* strains. This work provided evidence of the spread of this *bla*_KPC_-harboring untypeable plasmid in *Enterobacteriaceae* in China and highlights the urgent need for effective surveillance of KPC-producing *Enterobacteriaceae* to control rapid *bla*_KPC_ gene dissemination. A specific nomenclature for the untypeable plasmids is required.

## Author contributions

JH and ZZ were responsible for the study design and data interpretation. HD, YZ, XH, and JR collected all the clinical isolates and performed susceptibility tests. YS, GH, and RW carried out PCRs, transformation and conjugation experiments. JH performed RFLP and wrote the report. All authors revised, reviewed, and approved the final report.

### Conflict of interest statement

The authors declare that the research was conducted in the absence of any commercial or financial relationships that could be construed as a potential conflict of interest.

## References

[B1] Cai J. C. Zhou H. W. Zhang R. Chen G. X. (2008). Emergence of *Serratia marcescens, Klebsiella pneumoniae*, and *Escherichia coli* Isolates possessing the plasmid-mediated carbapenem-hydrolyzing beta-lactamase KPC-2 in intensive care units of a Chinese hospital. Antimicrob. Agents Chemother. 52, 2014–2018. 10.1128/AAC.01539-07 18332176 PMC2415814

[B2] Chen L. Chavda K. D. Fraimow H. S. Mediavilla J. R. Melano R. G. Jacobs M. R.. (2013). Complete nucleotide sequences of blaKPC-4- and blaKPC-5-harboring IncN and IncX plasmids from *Klebsiella pneumoniae* strains isolated in New Jersey. Antimicrob. Agents Chemother. 57, 269–276. 10.1128/AAC.01648-12 23114770 PMC3535970

[B3] Chen L. Hu H. Chavda K. D. Zhao S. Liu R. Liang H.. (2014). Complete sequence of a KPC-producing IncN multidrug-resistant plasmid from an epidemic *Escherichia coli* sequence type 131 strain in China. Antimicrob. Agents Chemother. 58, 2422–2425. 10.1128/AAC.02587-13 24395232 PMC4023777

[B4] Chen Y. T. Lin J. C. Fung C. P. Lu P. L. Chuang Y. C. Wu T. L.. (2014). KPC-2-encoding plasmids from *Escherichia coli* and *Klebsiella pneumoniae* in Taiwan. J. Antimicrob. Chemother. 69, 628–631. 10.1093/jac/dkt409 24123430

[B5] Christie P. J. (2004). Type IV secretion: the *Agrobacterium* VirB/D4 and related conjugation systems. Biochim. Biophys. Acta 1694, 219–234. 10.1016/j.bbamcr.2004.02.013 15546668 PMC4845649

[B6] Cuzon G. Naas T. Truong H. Villegas M. V. Wisell K. T. Carmeli Y.. (2010). Worldwide diversity of *Klebsiella pneumoniae* that produce beta-lactamase blaKPC-2 gene. Emerging Infect. Dis. 16, 1349–1356. 10.3201/eid1609.091389 20735917 PMC3294963

[B7] Gootz T. D. Lescoe M. K. Dib-Hajj F. Dougherty B. A. He W. Della-Latta P.. (2009). Genetic organization of transposase regions surrounding blaKPC carbapenemase genes on plasmids from *Klebsiella* strains isolated in a New York City hospital. Antimicrob. Agents Chemother. 53, 1998–2004. 10.1128/AAC.01355-08 19258268 PMC2681555

[B8] Ho P.-L. Li Z. Lo W.-U. Cheung Y.-Y. Lin C.-H. Sham P.-C. (2012). Identification and characterization of a novel incompatibility group X3 plasmid carrying blaNDM-1 in *Enterobacteriaceae* isolates with epidemiological links to multiple geographical areas in China. Emerg. Microbes Infect. 1, 39–45. 10.1038/emi.2012.37 PMC3630922 26038408

[B9] Hoffmann H. Roggenkamp A. (2003). Population genetics of the *Nomenspecies Enterobacter cloacae*. Appl. Environ. Microbiol. 69, 5306–5318. 10.1128/aem.69.9.5306-5318.2003 12957918 PMC194928

[B10] Huang J. Li X. Zhu N. Li G. (2012). Genetic characteristics of one highly multi-drug-resistant strain of *Klebsiella ozaenae*. J. Med. Microbiol. 61, 1303–1305. 10.1099/jmm.0.044115-0 22700546

[B11] Huang Y. Yu X. Xie M. Wang X. Liao K. Xue W.. (2016). Widespread dissemination of carbapenem-resistant *Escherichia coli* sequence type 167 strains harboring blaNDM-5 in clinical settings in China. Antimicrob. Agents Chemother. 60, 4364–4368. 10.1128/AAC.00859-16 27114282 PMC4914679

[B12] Jiang Y. Shen P. Wei Z. Liu L. He F. Shi K.. (2015). Dissemination of a clone carrying a fosA3-harbouring plasmid mediates high fosfomycin resistance rate of KPC-producing *Klebsiella pneumoniae* in China. Int. J. Antimicrob. Agents 45, 66–70. 10.1016/j.ijantimicag.2014.08.010 25450805

[B13] Jiang Y. Yu D. Wei Z. Shen P. Zhou Z. Yu Y. (2010). Complete nucleotide sequence of *Klebsiella pneumoniae* multidrug resistance plasmid pKP048, carrying blaKPC-2, blaDHA-1, qnrB4, and armA. Antimicrob. Agents Chemother. 54, 3967–3969. 10.1128/AAC.00137-10 20547789 PMC2934982

[B14] Monteiro J. Santos A. F. Asensi M. D. Peirano G. Gales A. C. (2009). First report of KPC-2-producing *Klebsiella pneumoniae* strains in Brazil. Antimicrob. Agents Chemother. 53, 333–334. 10.1128/AAC.00736-08 19015350 PMC2612176

[B15] Naas T. Cuzon G. Villegas M. V. Lartigue M. F. Quinn J. P. Nordmann P. (2008). Genetic structures at the origin of acquisition of the beta-lactamase bla KPC gene. Antimicrob. Agents Chemother. 52, 1257–1263. 10.1128/AAC.01451-07 18227185 PMC2292522

[B16] Nordmann P. Cuzon G. Naas T. (2009). The real threat of *Klebsiella pneumoniae* carbapenemase-producing bacteria. Lancet Infect. Dis. 9, 228–236. 10.1016/S1473-3099(09)70054-4 19324295

[B17] Pfeifer Y. Cullik A. Witte W. (2010). Resistance to cephalosporins and carbapenems in Gram-negative bacterial pathogens. Int. J. Med. Microbiol. 300, 371–379. 10.1016/j.ijmm.2010.04.005 20537585

[B18] Poirel L. Nordmann P. Lagrutta E. Cleary T. Munoz-Price L. S. (2010). Emergence of KPC-producing *Pseudomonas* aeruginosa in the United States. Antimicrob. Agents Chemother. 54:3072. 10.1128/AAC.00513-10 20421402 PMC2897310

[B19] Ribeiro T. G. Novais A. Branquinho R. Machado E. Peixe L. (2015). Phylogeny and comparative genomics unveil independent diversification trajectories of qnrB and genetic platforms within particular citrobacter species. Antimicrob. Agents Chemother. 59, 5951–5958. 10.1128/AAC.00027-15 26169406 PMC4576110

[B20] Robledo I. E. Aquino E. E. Sante M. I. Santana J. L. Otero D. M. Leon C. F.. (2010). Detection of KPC in *Acinetobacter* spp. in Puerto Rico. Antimicrob. Agents Chemother. 54, 1354–1357. 10.1128/AAC.00899-09 20038618 PMC2825984

[B21] Shen P. Wei Z. Jiang Y. Du X. Ji S. Yu Y.. (2009). Novel genetic environment of the carbapenem-hydrolyzing beta-lactamase KPC-2 among *Enterobacteriaceae* in China. Antimicrob. Agents Chemother. 53, 4333–4338. 10.1128/AAC.00260-09 19620332 PMC2764158

[B22] Shen P. Zhang Y. Li G. Jiang X. (2016). Characterization of the genetic environment of the blaKPC-2 gene among *Klebsiella pneumoniae* isolates from a Chinese Hospital. Braz. J. Infect. Dis. 20, 384–388. 10.1016/j.bjid.2016.04.003 27183358 PMC9427567

[B23] Thomson G. K. Snyder J. W. McElheny C. L. Thomson K. S. Doi Y. (2016). Coproduction of KPC-18 and VIM-1 Carbapenemases by Enterobacter cloacae: implications for newer beta-lactam-beta-lactamase inhibitor combinations. J. Clin. Microbiol. 54, 791–794. 10.1128/JCM.02739-15 26719440 PMC4767958

[B24] Wayne P. (2017). Performance Standards for Antimicrobial Susceptibility Testing [S]: Twenty-Seventh Informational Supplement. Pennsylvania, PA: Clinical and Laboratory Standards Institute, M100–S27.

[B25] Yigit H. Queenan A. M. Anderson G. J. Domenech-Sanchez A. Biddle J. W. Steward C. D.. (2001). Novel carbapenem-hydrolyzing beta-lactamase, KPC-1, from a carbapenem-resistant strain of *Klebsiella pneumoniae*. Antimicrob. Agents Chemother. 45, 1151–1161. 10.1128/AAC.45.4.1151-1161.2001 11257029 PMC90438

[B26] Zhang R. Liu L. Zhou H. Chan E. W. Li J. Fang Y.. (2017). Nationwide surveillance of clinical carbapenem-resistant enterobacteriaceae (CRE) strains in China. EBioMed. 19, 98–106. 10.1016/j.ebiom.2017.04.032 28479289 PMC5440625

[B27] Zhang R. Zhou H. W. Cai J. C. Chen G. X. (2007). Plasmid-mediated carbapenem-hydrolysing beta-lactamase KPC-2 in carbapenem-resistant *Serratia marcescens* isolates from Hangzhou, China. J. Antimicrob. Chemother. 59, 574–576. 10.1093/jac/dkl541 17251347

[B28] Zhang Y. Wang Q. Yin Y. Chen H. Jin L. Gu B.. (2018). Epidemiology of carbapenem-resistant enterobacteriaceae infections: report from the China CRE network. Antimicrob. Agents Chemother. 62:e01882–17. 10.1128/AAC.01882-17 29203488 PMC5786810

[B29] Zheng B. Huang C. Xu H. Yu X. Zhang J. Wang X.. (2018). Complete nucleotide sequences of two KPC-2-encoding plasmids from the same *Citrobacter freundii* isolate. J. Antimicrob. Chemother. 73, 531–533. 10.1093/jac/dkx381 29092035

