# Supplementary material for: Further Spread of a blaKPC-Harboring Untypeable Plasmid in Enterobacteriaceae in China
Source: Front Microbiol. 2018 Aug 21;9:1938. doi: 10.3389/fmicb.2018.01938 (PMC6111213; doi:10.3389/fmicb.2018.01938)
Supplement: Supplementary file 1 [file Table_1.DOCX]

**Supplementary S1**

According to previous studies ([Hoffmann and Roggenkamp, 2003](#_ENREF_1); [Ribeiro et al., 2015](#_ENREF_2)), comparative analysis of the *RecN* sequences to type strains classified CP40, CF111 and CK61 into *Citrobacter* sp. I, *Citrobacter freundii* and *Citrobacter koseri* groups, and with the highest similarity to *Citrobacter portucalensis* A60T, *Citrobacter freundii* strain S6-11 and *Citrobacter koseri* ATCC BAA-895, respectively (Fig S1). Comparison of the *Hsp60* revealed highest similarity of EAK7 to *Enterobacter asburiae* ATCC 35953 (Fig S1).







A

B

Fig S1. Neighbor-joining (NJ) tree based on the comparison of *recN* (A) and *Hsp60* (B) gene sequences of the *Citrobacter* species and *Enterobacter asburiae* respectively. Bootstrap was 1,000 replications and cut-off value for condensed tree was 80%. Horizontal bar, genetic distance of 0.05. CP40, CF111, CK61 and EAK7 were marked with black [square](javascript:;).

**References:**

Hoffmann, H., Roggenkamp, A. (2003). Population Genetics of the Nomenspecies Enterobacter cloacae. *Applied and Environmental Microbiology, 69*, 5306-5318.doi:10.1128/aem.69.9.5306-5318.2003

Ribeiro, T. G., Novais, A., Branquinho, R., Machado, E., Peixe, L. (2015). Phylogeny and Comparative Genomics Unveil Independent Diversification Trajectories of qnrB and Genetic Platforms within Particular Citrobacter Species. *Antimicrob Agents Chemother, 59*, 5951-5958.doi:10.1128/AAC.00027-15
