# Supplementary material for: Further Spread of a blaKPC-Harboring Untypeable Plasmid in Enterobacteriaceae in China
Source: Front Microbiol. 2018 Aug 21;9:1938. doi: 10.3389/fmicb.2018.01938 (PMC6111213; doi:10.3389/fmicb.2018.01938)
Supplement: Supplementary file 2 [file Table_2.DOCX]

Supplementary S2

Pulsed-field gel electrophoresis (PFGE) was performed according to the previous study ([Huang et al., 2016](#_ENREF_1)). The genome DNAs of *E. coli* EC84 and EC86 strains were extracted and digested by XbaI. Electrophoresis parameters were shown in following table.

| Conditions |  |
| --- | --- |
| Run Time | 18.5 hr |
| Initial Switch Time | 6.75 sec |
| Final Switch Time | 35.38 sec |
| Voltage Gradient | 6 V/cm |
| Included Angle | 120° |
| Ramping | Linear |
| Intial current (mA) | 137 mA |


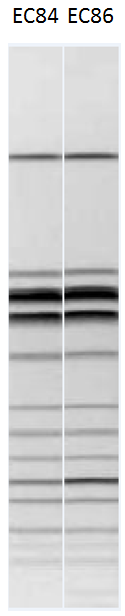


Supplementary S2. PFGE results of E. coli strains EC84 and EC86. Identical PFGE patterns were obtained via XbaI digestion.

Huang, Y., Yu, X., Xie, M., Wang, X., Liao, K., Xue, W., et al. (2016). Widespread Dissemination of Carbapenem-Resistant Escherichia coli Sequence Type 167 Strains Harboring blaNDM-5 in Clinical Settings in China. *Antimicrob Agents Chemother, 60*, 4364-4368.doi:10.1128/AAC.00859-16
